# CEST MRI detects antiretroviral drug toxicities in the developing mouse brain

**DOI:** 10.3389/fphar.2025.1681094

**Published:** 2025-10-29

**Authors:** Micah Summerlin, Mariano G. Uberti, Dhananjay Shinde, Emma G. Foster, Brady Sillman, Manjeet Kumar, Baojin Yao, Dongming Peng, Benson J. Edagwa, Howard E. Gendelman, Yutong Liu, Aditya N. Bade

**Affiliations:** ^1^ Department of Pharmacology and Experimental Neuroscience, University of Nebraska Medical Center, Omaha, NE, United States; ^2^ Department of Radiology, University of Nebraska Medical Center, Omaha, NE, United States; ^3^ Department of Pathology, Microbiology and Immunology, University of Nebraska Medical Center, Omaha, NE, United States; ^4^ Department of Electrical and Computer Engineering, University of Nebraska – Lincoln, Lincoln, NE, United States; ^5^ Department of Pharmaceutical Sciences, University of Nebraska Medical Center, Omaha, NE, United States

**Keywords:** CEST MRI, dolutegravir, HIV-1, long-acting antiretroviral formulation, neurodevelopment

## Abstract

Advances in antiretroviral drugs (ARVs) have achieved remarkable success in preventing gestational human immunodeficiency virus type 1 (HIV-1) transmission from mother to fetus. This is reflected in the rising numbers of HIV-1-exposed uninfected (HEU) children. Worldwide, the number of HEU children exceeds sixteen million, with more than one million children joining this group each year. Although HEU children remain uninfected, they are at an increased risk of neurodevelopmental deficits. Notably, *in utero* exposure to HIV-1 and ARVs is a causative factor. Both are linked to adverse neurodevelopment, warranting close clinical monitoring and therapeutic intervention. We now demonstrate that chemical exchange saturation transfer (CEST) MRI can be used to successfully monitor *in utero* ARV-exposure-associated embryo brain metabolomic and macromolecular dysregulations in a mouse model. CEST hyperintensities at −3.5 ppm (nuclear Overhauser effect) and 3.5 ppm (amide/amine protons) are measured in the brains of mouse embryos exposed to dolutegravir (DTG). These reflect DTG-induced alterations in cellular membrane lipids, mobile proteins or peptides, and glutamate levels. All demonstrate impaired neuronal development. Non-targeted metabolomics confirms the CEST results. These support the observations of DTG-induced differential expression of lipids and metabolites that reflect deficits in energy production, cell metabolism, post-translational protein modifications, and transport pathways. Furthermore, CEST MRI demonstrated the therapeutic benefits of long-acting nanoformulation delivery of DTG in mitigating neurodevelopmental impairments. These data, taken together, support the utility of CEST MRI as a non-invasive imaging biomarker for detecting neurodevelopmental deficits.

## Introduction

The number of children born to mothers living with human immunodeficiency virus type 1 (LWH) has steadily increased. Each year, more than a million women LWH give birth while on antiretroviral therapy (ART) (Ramokolo et al., 2019; Crowell et al., 2020; Foster et al., 2022). This is due to the growing availability of antiretroviral drugs (ARVs). At the same time, improved HIV-1 detection remains operative in both resource-rich and resource-limited countries (RRCs and RLCs). In 2023, more than 80% of pregnant women LWH, worldwide, had access to ART (UNAIDS, 2024). Prior to ART, up to 40% of infants contracted HIV-1 through vertical transmission from mothers LWH. ART has significantly reduced vertical HIV-1 transmission to less than 1% (Peters et al., 2017; CDC, 2018; Schnoll et al., 2019; Rasi et al., 2022). While ART prevents mother-to-child HIV-1 transmission and improves maternal health, concerns remain about adverse effects of *in utero* ARVs exposure on fetal development. This includes ART-related effects on pre- and post-natal neurodevelopment (Hill et al., 2018; Schnoll et al., 2019; Bade AN et al., 2021; Foster et al., 2022), which may include physiological, metabolic, or functional changes in the developing brain. This is underscored by noted risks of postnatal neurodevelopmental deficits in HIV-1-exposed uninfected (HEU) children following *in utero* ARV exposure to efavirenz (EFV), atazanavir (ATV), didanosine (ddI), or dolutegravir (DTG) (Sirois et al., 2013; Caniglia et al., 2016; Cassidy et al., 2019; Crowell et al., 2020; Wedderburn et al., 2022; Bulterys et al., 2023). However, these assessments require longitudinal studies of functional neurodevelopment after birth. Furthermore, the lack of neuroimaging biomarkers for monitoring fetal brain development during gestation limits the opportunity for early detection and therapeutic interventions. With significant ongoing efforts to develop new ARVs with high potency, strong viral resistance activity, and longer pharmacokinetic (PK) profiles, such as long-acting cabotegravir and rilpivirine (LA CAB and RPV) and lenacapavir (LEN), concerns about gestational toxicity would persist (FDA, 2021a; FDA, 2021b; Gilead Sciences, 2022). Therefore, developing a novel non-invasive bioimaging tool to detect early signs of gestational ARV exposure-associated adverse effects on neurodevelopmental cellular processes is timely. Such a bioimaging tool will define ARVs or a class thereof that produce adverse effects on neurodevelopment irrespective of their classification, administration route, or PK profile.

Herein, we aim to develop a novel magnetic resonance imaging (MRI) tool to non-invasively monitor DTG-associated neurodevelopmental adverse events during gestation in a rodent model. DTG was chosen as the drug of interest due to its universal utilization as a part of first-line ART and its potential link to increased risk of neurodevelopmental deficits following periconceptional usage (Zash et al., 2018b; Cabrera et al., 2019; Zash et al., 2019; Crowell et al., 2020; Mohan et al., 2020; Bade AN et al., 2021; Foster et al., 2023; Zizioli et al., 2024). The MRI technique of interest is known as chemical exchange saturation transfer (CEST). It relies on the intrinsic chemical properties of tissue-resident biomolecules (metabolites, mobile proteins, and peptides) (Ward et al., 2000; van Zijl and Yadav, 2011; Vinogradov et al., 2013; Wu et al., 2016; Pankowska et al., 2019). The CEST signal is detected when an exchangeable proton of a biomolecule is magnetically saturated and transferred to water by chemical exchange causing reduction in signal. Continuous exchange with bulk water protons amplifies this signal reduction, enabling the detection of the signature biomolecule at low concentrations (Ward et al., 2000; van Zijl and Yadav, 2011; Vinogradov et al., 2013; Wu et al., 2016; Pankowska et al., 2019). Compared to traditionally utilized proton magnetic resonance spectroscopy (^1^H-MRS), CEST MRI offers greater sensitivity and spatial resolution for metabolite detection. Moreover, external contrast agents are not required. This study successfully demonstrated that CEST MRI detects DTG-induced neuronal impairments. In replicate studies, CEST MRI hyperintensities were observed at −3.5 ppm [nuclear Overhauser effect (NOE)] and at 3.5 ppm (amide and amine protons). These findings indicate adverse effects on membrane lipids, mobile proteins or peptides, and the neurotransmitter, glutamate resulting from *in utero* DTG exposure (Wu et al., 2016; Zhang et al., 2018; Jia et al., 2020; Zhao et al., 2023; Guo and Peng, 2025). Moreover, CEST MRI also demonstrated the benefits of a poloxamer-based long-acting formulation of DTG (NDTG) in preventing gestational drug exposure-induced adverse changes in lipids and metabolites, which are linked to impaired neuronal development. Based on these preliminary data, we propose that CEST MRI could be developed to detect ART-linked fetal neuropathological biomarkers.

## Materials and methods

### Animals and study approvals

All animal studies included in the manuscript were approved by the University of Nebraska Medical Center (UNMC) Institutional Animal Care and Use Committee (IACUC) in accordance with the standards of the Guide for the Care and Use of Laboratory Animals (National Research Council of the National Academies, 2011). C3H/HeJ mice (male and female, 10–12 weeks of age), were purchased from the Jackson Laboratory (Bar Harbor, ME) and housed at UNMC within microisolator cages in climate-controlled laboratory animal facilities. Mice were given free access to irradiated rodent feed (TD. 7012, Envigo Teklad diet, Madison, WI) and sterile water. Animals were maintained on a 12-h light-dark cycle and were acclimated to the animal facility environment for approximately 1–2 weeks before experiments. Healthy animals displaying normal behavior, movement, and weight were utilized for all experiments. For timed mating, a single female was placed with a single male overnight and checked next morning for a positive vaginal plug. Females identified with vaginal plugs were weighed and randomly distributed to treatment (DTG or NDTG) or control (vehicle) group. Male mice were utilized only for mating. Male mice did not receive any drug or chemical treatment and were not utilized for any tests. The time of conception was considered midnight on the night of the mating. Thus, treatment of plugged females was initiated on the day of the positive vaginal plug detection, and this day was identified as gestation day (GD) 0.5.

### Study groups

For the first study to assess the sensitivity of CEST MRI in evaluating drug-induced alterations in embryo brain metabolites, pregnant mice (C3H/HeJ) determined by positive vaginal plugs were randomly distributed in two groups. In group one, pregnant mice (dams) were treated orally every day with native DTG at ten times the human therapeutic equivalent dosage (50 mg/kg, mouse weight equivalent; n = 5) by oral gavage starting at GD 0.5 up to 16.5. In group two, dams were treated orally every day with the vehicle (n = 5) utilized to prepare DTG solution for oral administration [Control; dimethylsulfoxide:Solutol^®^:50 mM *N*-methylglucamine in 3% mannitol (1:1:8, v:w:v)] by oral gavage. The administration scheme and volume per administration for the vehicle was similar to native DTG oral administration.

In a similar paradigm, for the second study conducted for rigor and reproducibility, pregnant mice (C3H/HeJ) identified by positive vaginal plugs were randomly distributed to three treatment groups. In the first treatment group, dams were treated orally every day with native DTG at human therapeutic equivalent dosage (5 mg/kg, mouse weight equivalent; n = 5) by oral gavage starting at GD 0.5 up to GD 16.5. In the second treatment group, dams were treated orally every day with the vehicle (n = 8) utilized to prepare DTG solution for oral administration by oral gavage. The vehicle composition was the same as described above for the first study. In the third treatment group, dams were injected with a single intramuscular (IM) injection of NDTG (45 mg/kg, mouse weight equivalent; n = 4) at GD 0.5.

Women who used DTG during the periconceptional period faced a potential risk of adverse pregnancy outcomes compared to those who only received the drug during pregnancy (Zash et al., 2018a; Zash et al., 2018b; Zash et al., 2019). Thus, DTG administration was initiated on the day pregnancy was confirmed, indicated by a positive vaginal plug (GD 0.5) in both of our studies. This drug administration approach also ensured that the drug exposure levels remained consistent across all pregnant dams. Regarding the NDTG group, we used a single IM injection of 45 mg/kg dosage at GD 0.5. In previous studies, we observed that a single IM dose of NDTG administered at a 45 mg/kg sustains therapeutic drug levels for up to a month in mice (Sillman et al., 2018; Foster et al., 2023). Moreover, the benefits of this dosage and formulation type of NDTG in preventing developmental neuronal toxicity induced by orally administered DTG at human therapeutic equivalent dosages were noted (Foster et al., 2023).

For each study, weight gain in dams during gestation was measured. Dams were humanely euthanized at GD 17.5, and embryos and placentas were harvested for PK/BD or biological evaluations. Effects of vehicle, oral native DTG or IM injectable NDTG exposure on mice embryo development were evaluated. At the time of harvest, the total number of implants (litter size, resorption rates or viable embryos) was recorded for each pregnancy and embryos were assessed for neural tube defects (NTDs). Further, whole brain tissues from normal viable embryos were processed for tissue DTG concentrations or global metabolomic evaluations.

### CEST MRI

All MRI data were acquired using a Bruker^®^ PharmaScan 7.0 T/16 cm small animal scanner (Bruker, Billerica, MA), operating at 300.41 MHz using Bruker mouse body coil. CEST MRI data were acquired using a rapid acquisition with relaxation enhancement (RARE) sequence with optimized parameters: saturation RF amplitude = 2 μT and duration = 1 s. We implemented a non-uniform frequency sampling approach to comprehensively capture the Z-spectrum while maintaining high resolution in regions of interest. Specifically, frequency offsets were sampled from −5 to 5 ppm with a step size of 0.2 ppm in the peripheral ranges [-5 ppm, −4 ppm] and [4 ppm, 5 ppm], and a finer step size of 0.1 ppm in the central range [-4 ppm, 4 ppm]. This sampling strategy provided optimal characterization of key CEST contrasts of interest at 2 ppm, 3.5 ppm, and −3.5 ppm (relayed nuclear Overhauser effect, rNOE). Data were acquired from a single axial slice positioned at the center of the embryo brain with the following parameters: slice thickness = 1 mm, matrix = 128 × 128, and field of view (FOV) = 35 (left-right) × 25 (dorsal-ventral) mm^2^. To correct for B_0_ field inhomogeneities, we employed the Water Saturation Shift Referencing (WASSR) method (Kim et al., 2009), which is essential for accurate quantification of CEST effects. For data analysis, we implemented a 5-pool Lorentzian fitting approach to isolate and quantify specific CEST contrasts. This method enabled us to differentiate the target CEST signals (2 ppm/creatine, 3.5 ppm/mobile proteins/peptides and glutamate, −3.5 ppm/rNOE) from confounding effects including magnetization transfer contrast (MTC) at −1.5 ppm and direct water saturation at 0 ppm, ensuring accurate assessment of metabolite-specific CEST effects. For data inclusion criteria CEST MRI data were evaluated for image artifacts including motion, ghosting, and Gibbs ringing. Datasets exhibiting severe artifacts were excluded. The quality of Lorentzian model fitting to the Z-spectra was assessed using the coefficient of determination (R^2^). Data with poor model fit (R^2^ < 0.8) was excluded from the analysis.

### Proton magnetic resonance spectroscopy (^1^H-MRS)

MR images were acquired for anatomical reference using a multislice RARE sequence (Effective TE = 24 ms, Rare Factor = 8, TR = 2000 ms, Number of Averages = 6, Scan Time = 3 min 50 s; FOV = 35 × 25 mm^2^, Matrix Size = 256 × 192, Spatial Resolution = 0.137 × 0.130 mm^2^, Number of Slices = 15, Slice Orientation = axial, Slice Thickness = 0.5 mm). All first and second-order shim terms were first automatically adjusted in the VOI using MAPSHIM^®^ (Bruker, Billerica, MA), with a final shim, if necessary, performed manually to achieve a water line width of 10–15 Hz. The water signal was suppressed by variable power RF pulses with optimized relaxation delays (VAPOR). ^1^H-MRS data sets were obtained using semiLASER localization with timing parameters (TE/TR = 40/4000 ms, 576 averages, spectral width = 3,005 Hz, 2048 points) from a 3 × 3 × 2.5 mm^3^ (22.5 μL) volume of interest located in the ventricular/sub-ventricular zones and adjacent cortex. The acquisition time was approximately 1 h per data set. For each experiment, one data set was acquired without water suppression to be used as the water concentration reference for the quantitation process. Unsuppressed water spectra were obtained with identical metabolite spectra parameters except for the following: TR = 1 s, number of averages (NA) = 1, and Receiver Gain = 64. One 64 average (for quality assessment) plus four 128-average data sets were acquired for metabolite measurements using VAPOR scheme for water suppression. The protocol followed best practices for preclinical MRS (Lanz et al., 2020). For the MRS data analysis, pre-processing was done automatically using a home-built software programmed in Matlab (MathWorks, Natick, MA). *Free induction decay (FID)* signals were first transformed to the frequency domain using Fourier Transformation and spectra of the signals were obtained. Then, a *truncation* operation was used to remove the leading 68 points of the FID which correspond to zero data introduced by the digital electronics of the system before the meaningful data stream. This was followed by a *zero filling* operation to add back 68 zero-value points at the end of the *fid* in order to maintain the signal with the 2048 original number of points. Then, a 5 Hz *apodization* operation was applied in order to reduce noise and truncation artifacts. All spectra were then zero-order phased and first-order phased using a time of −0.22 ms. Finally, the FID(s) from the water-unsuppressed acquisitions were corrected for frequency shifts using the NAA peak from the basis sets as reference and then summed for the metabolite concentration measurement. After all pre-processing was done, the water-unsuppressed “water” spectra and the water-suppressed “metabolite” spectra were then submitted to LCModel (LCMODEL Inc., CA) for quantitation. Concentration values of individual metabolites reported in institutional units (i.u.) were output in spreadsheet for statistical analysis (Provencher, 1993; Naressi et al., 2001; Wijnen et al., 2010). Furthermore, the following data inclusion criteria was employed for rigorous assessments. Spectral quality control was performed on all datasets. Spectra were included in the analysis if they met criteria of signal-to-noise ratio (SNR) ≥ 12 and water peak linewidth (LW) ≤ 0.1 ppm. For metabolite quantification reliability, inclusion of a metabolite in the statistical analysis required a mean Cramer-Rao Lower Bound (CRLB) ≤ 30% across subjects within a group. Additionally, individual metabolite measurements were considered reliable if the CRLB was ≤20%. Only one dataset was excluded from further analysis due to poor spectral quality attributed to subject movement.

### LC-HRMS/MS analysis of metabolites

After CEST MRI scanning, dams were humanely euthanized and whole embryo brains were isolated. Embryo brain tissues were flash frozen in liquid nitrogen and stored in −80 °C. Later, embryo whole brain tissues were homogenized and processed for non-targeted metabolite analysis as previously described (Van Roy et al., 2024). Five embryo brains per group (control or DTG 5 mg/kg/day) were selected. Each embryo tissue was from a different dam for both groups. Non-targeted metabolite analysis was performed using High-resolution mass spectrometer Orbitrap viz., Exploris 480 (Thermo, USA) connected with ultra-high performance liquid chromatography (UHPLC) viz., Vanquish system procured from Thermo, USA. The metabolite separation was performed by liquid chromatography using XBridge Amide (150 × 2.1 mm ID; 1.7 µm particle size, Waters, USA) analytical column and a binary solvent system which was infused at a flow rate of 0.3 mL/min. A guard XBridge Amide column (20 × 2.1 mm ID; 3.5 µm particle size, Waters, USA) was connected in front of analytical column. Mobile phase A was composed of 10 mM ammonium acetate, 10 mM ammonium hydroxide containing 5% acetonitrile in LC-MS grade water; mobile phase B was 100% LC-MS grade acetonitrile. pH of the mobile phase A was adjusted to 8.0 using glacial acetic acid. The UHPLC pumps were operated in gradient mode. Amide column was maintained at 40 °C and autosampler temperature was maintained at 5 °C throughout during data acquisition. Injection volume for all samples was 5 µL. ^13^C^15^N-labeled canonical amino acid mix was used as the internal standard. HRMS Orbitrap (Exploris 480) operated in polarity switching mode was used for untargeted metabolomics in data-dependent MS/MS acquisition mode (DDA). Electrospray ionization (ESI) parameters were optimized and are as follows: electrospray ion voltage of −2,700 V and 3,500 V in negative and positive mode respectively, ion transfer tube temperature was maintained at 300 °C and vaporizer temperature was 200 °C. Data was acquired in the m/z scan range was 70–1,050 Da. Orbitrap resolution for precursor ion as well as for fragment ion scan were maintained at 240,000 and 120,000 respectively. Normalized collision energies at 30%, 50% and 150% were used for the fragmentation. Data was acquired in profile mode. Identification and detection of all metabolites was aided by the Compound Discoverer (CD) software procured from Thermo USA. The HMDB and KEGG databases plugged-in with CD software were used for metabolite identifications. Various plots such as volcano and PCA plots were generated using MetaboAnalyst 6.0. Identified metabolites were analyzed for biological processes and functional pathways using MetaboAnalyst 6.0 and Ingenuity Pathway Analysis (IPA). Heatmaps were generated using GraphPad Prism 10 software (La Jolla, CA).

### Nanoparticle preparation and characterization

NDTG was prepared in endotoxin-free water, pH 7.0 at a ratio of 10:1 (w/w) drug:poloxomer 407 (P407; Sigma-Aldrich, St. Louis, MO, USA). Starting drug concentration was 5% (w/v). HyPure endotoxin-free cell culture grade water was purchased from Cytiva (Marlborough, MA, USA). This pre-suspension was homogenized on an Avestin EmulsiFlex-C3 high-pressure homogenizer (Ottawa, ON, Canada) at 20,000 ± 1,000 PSI with the chiller set to 6 °C to form the desired particle size. Nanoparticles were characterized for hydrodynamic particle diameter (size), polydispersity indices (PDI), and zeta potential using a Malvern Zetasizer Nano-ZS (Worcestershire, UK). For quality control, selection criteria were set to nanoformulations with size 200–500 nm, PDI at 0.25 ± 0.05, and encapsulation efficiency greater than 70%. Nanoformulations with these selection criteria achieved stabilization of particles and extended pharmacokinetics (PK) profile in rodent models (Sillman et al., 2018; Kulkarni et al., 2020; Deodhar et al., 2022). Scanning electron microscopy (SEM) was performed for morphological assessments of nanoparticles (Sillman et al., 2018). All formulations were suitably syringable and non-viscous for readily passing through a 28 G needle. In addition, these nanoparticles were analyzed for the drug content using UPLC-TUV by extracting drug in HPLC-grade methanol (1,000-fold dilutions). A Waters ACQUITY UPLC H-Class system with tunable ultraviolet/visible (TUV) detector and Empower 3 software were used to measure drug concentrations of the nanoformulation. A Phenomenex Kinetex 5 μm C18 column (150 × 4.6 mm) (Torrance, CA, USA) was utilized to separate DTG. Later, using isocratic elution with a mobile phase consisting of 65% 50 mM potassium phosphate monobasic (KH_2_PO_4_), pH 3.2/35% HPLC-grade Acetonitrile (ACN) at a flow rate of 1.0 mL/min, DTG was detected at 254 nm. Reagents for these HPLC tests including HPLC-grade methanol, KH_2_PO_4_, and ACN were purchased from Fisher Scientific (Waltham, MA, USA). DTG content was determined relative to peak areas of drug standards (0.05–50 μg/mL) in methanol. Encapsulation efficiency (EE%) was calculated using the following formula. Encapsulation efficiency (EE%) = (Drug_encapsulated in formulation_ [mg]/Drug_total added to premix_ [mg]) x 100%.

### DTG PK and biodistribution (BD) tests

PK and BD profiles of oral native DTG (therapeutic or supratherapeutic) administration or IM injectable NDTG were determined. From dams, blood samples were collected by cheek puncture (submandibular vein) using a 5 mm lancet (MEDIpoint, Inc., Mineola, NY) at GD 8.5 and by terminal heart puncture using a 28 G insulin syringe (Exelint International Co., Redondo Beach, CA) at GD 17.5. Blood samples were collected in heparinized tubes. Later, plasma was collected from blood by centrifuging heparinized tubes at 2,000 × g for 8 min for the quantitation DTG concentrations. Biodistribution of DTG to embryo brain or placenta was determined at GD 17.5. According to our previous publications, concentrations of DTG were quantitated in dams’ plasma, and placenta or embryo brain tissue homogenates by ultra-performance liquid chromatography tandem mass spectrometry (UPLC-MS/MS) using a Waters ACQUITY Premier UPLC (Milford, MA, USA) connected to a Xevo TQ-XS mass spectrometer and analyzed using Waters MassLynx V4.2 software (Milford, MA, USA) (Sillman et al., 2018; Bade AN et al., 2021; Deodhar et al., 2022). Each plasma or tissue sample was randomly selected from a distinct litter for measuring DTG levels. All solvents for sample processing and UPLC-MS/MS analysis were Optima grade (Fisher Scientific). For quantitation of DTG concentrations, chromatographic separation of a 10 μL sample injection was performed on a Waters ACQUITY UPLC BEH Shield RP18 column (1.7 μm, 2.1 mm × 100 mm) at 25 °C using a 10-min gradient method. Mobile phase A contained 7.5 mM ammonium formate in water, adjusted to pH 3 using formic acid. Mobile phase B contained 100% ACN. For the first 5 min, the mobile phase composition was 40% B. Afterwards, a cleaning step consisting of 95% B was held constant for 3 min before equilibrating the column back to initial conditions for 2 min. Capillary voltage of 3.3 kV was used for DTG and DTG-d3 multiple reaction monitoring (MRM) transitions of 420.16 > 277.17 and 423.18 > 135.96 using collision energies of 26 eV and 54 eV and cone voltages of 6 V and 28 V, respectively. Drug concentration quantitation was assessed using analyte peak area to internal standard peak area ratios.

### Statistical analysis

GraphPad Prism 10 software (La Jolla, CA) was utilized to perform statistical analyses. For all results, data were expressed as mean ± standard error of the mean (SEM) with a minimum of 3 biological replicates. For comparisons between two groups, student’s t test (two-tailed) was used. A one-way or two-way ANOVA followed by Tukey’s test was utilized to compare three or more groups. Data inclusion or exclusion criteria for each parameter were explained above in individual method sections. For all data sets, statistical significance was designated as ^#^p < 0.1, *p < 0.05, **p < 0.01, ***p < 0.001, ****p < 0.0001.

## Results

### Embryo brain DTG exposures

**FIGURE 1:**
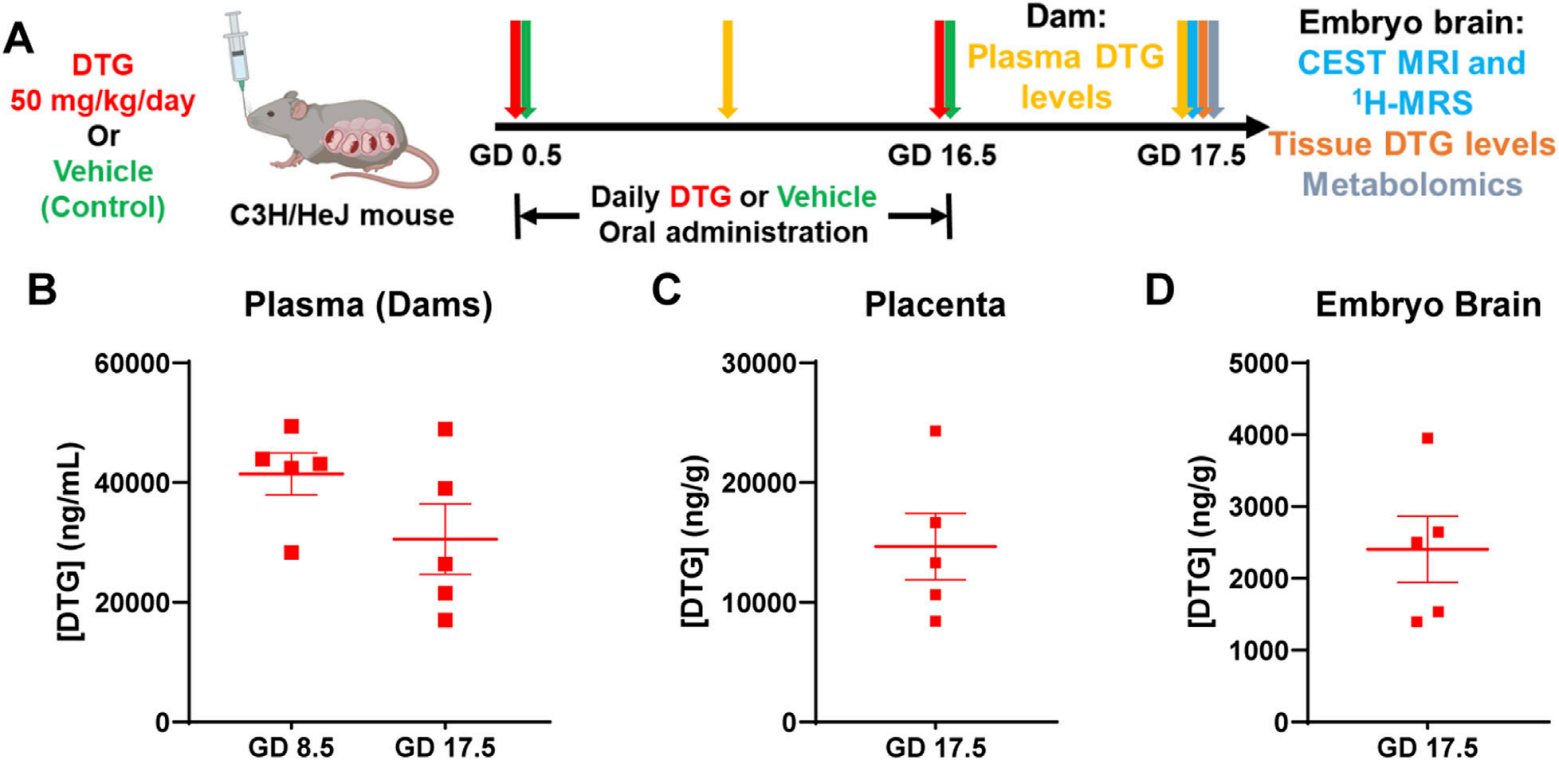
PK and BD profiles of DTG. **(A)** Schematic representation of timeline and study design. Pregnant C3H/HeJ female mice (dams) were randomly distributed in two study groups. In group one, dams were daily administered native DTG at 50 mg/kg dose (mouse weight) by oral gavage starting from gestation day (GD) 0.5 to GD 16.5. In group two, dams were daily administered with the vehicle by oral gavage starting from GD 0.5 to 16.5 (control group). CEST MRI and embryo brain harvesting for DTG concentrations and metabolomic tests were conducted on GD 17.5. **(B)** DTG concentrations in plasma of dams at GD 8.5 and 17.5. **(C)** DTG concentrations in placental tissue at GD 17.5. **(D)** DTG concentrations in embryo brain tissue at GD 17.5 **(B–D)** Each plasma or tissue sample represents a distinct litter. Data are expressed as mean ± SEM, N = 5 animals.

To determine if CEST MRI can be used to detect DTG-linked metabolomic alterations in embryo brain, pregnant dams (C3H/HeJ) were evaluated in two study groups, DTG or vehicle (control) (Figure 1A). Firstly, body weight gains of dams during pregnancy were recorded. No significant differences were observed between control and DTG groups (Supplementary Figure 1). Later, PK and BD profiles of the DTG were assessed to confirm embryo brain drug exposure (Figures 1A–D). Following oral administration of a DTG at supratherapeutic dose, approximately ten times the relevant human DTG concentrations were found in the dams’ plasma. The average plasma drug concentrations were 41,420 ± 3,505.30 and 30,560 ± 5,877.30 ng/mL at GD 8.5 and 17.5, respectively (Figure 1B). DTG levels in the placenta at GD 17.5 were recorded at 14,671.30 ng/g (Figure 1C). Notably, DTG concentrations in embryonic brain tissue at GD 17.5 measured at 2,405.60 ± 460.35 ng/g (Figure 1D). The ratio of embryo brain to dam plasma DTG levels was 0.07, and the ratio of placenta tissue to dam plasma was 0.48 at GD 17.5. The ratio of embryo brain to placenta DTG level was 0.16. These ratios of drug levels are consistent with previous findings when a supratherapeutic dose was administered (Bade AN et al., 2021). Overall, PK and BD data confirm *in utero* DTG exposure of the embryo brain.

### CEST MRI measures DTG-induced embryo brain metabolites

**FIGURE 2:**
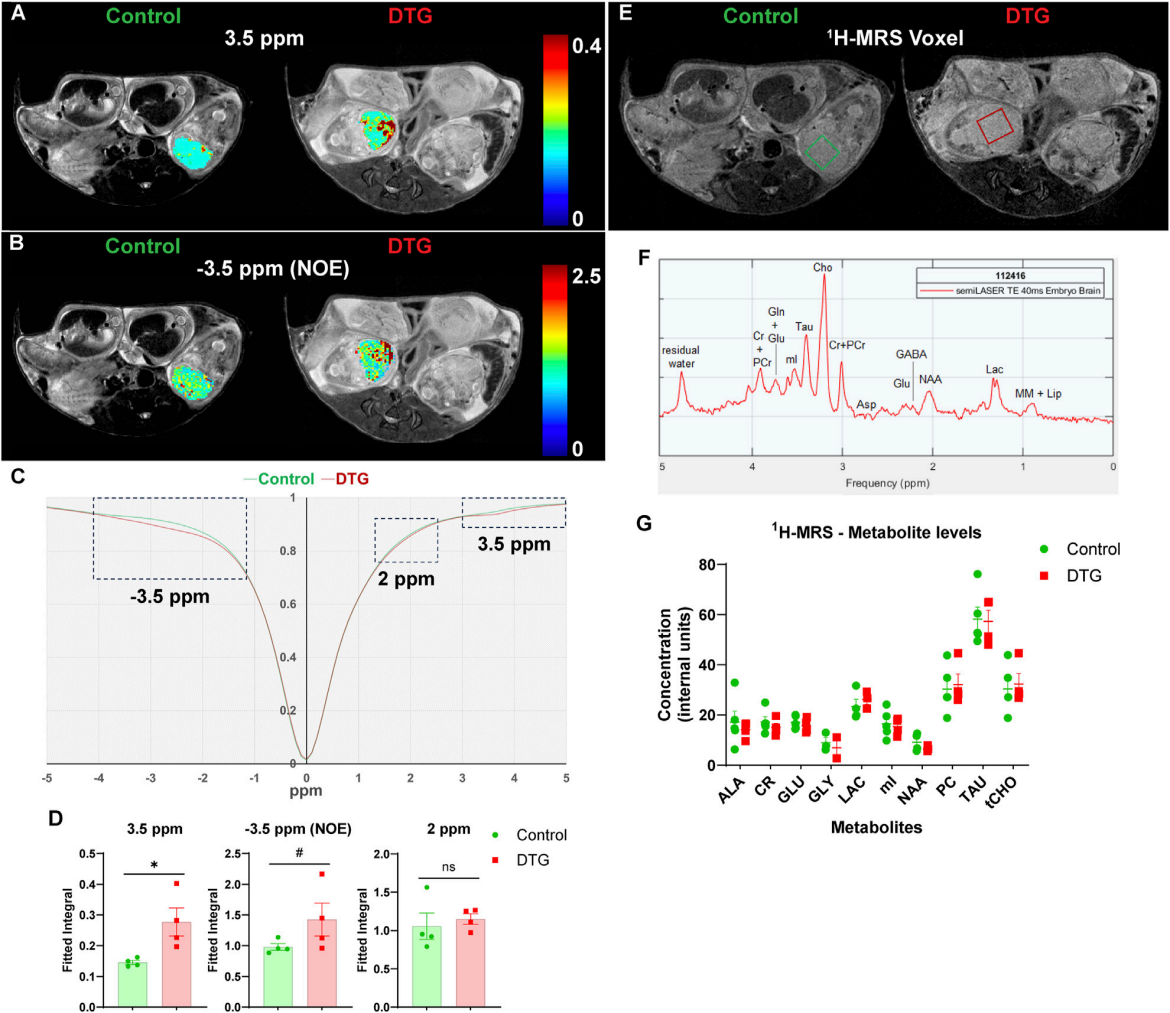
CEST MRI identifies DTG-induced changes in embryo brain metabolites. Live pregnant dams (C3H/HeJ) were scanned at GD 17.5 using a 7T scanner. **(A,B)** Pixel-by-pixel integral maps of fitted CEST effect. Hyperintensities were detected in DTG-treated embryo brains at 3.5 ppm and −3.5 ppm **(C)** Z-spectra of controls and DTG-treated mice. **(D)** Group comparisons of fitted integrals of CEST effect at 2 ppm, 3.5 ppm and −3.5 ppm on embryo brain. N = 4 animals/group. T-test (two-tailed) was used (^#^P < 0.1, *P < 0.05; ns = not significant). **(E-G)** ^1^H-MRS analyses. **(E,F)** Representative ^1^H-MRS voxels and spectrum presenting metabolites from mouse embryo brain. **(G)** Metabolite levels (mean ± SEM) expressed as internal units (i.u.) from ^1^H-MRS scans of embryo brains of control and DTG groups. N = minimum 3 animals/group. T-test (two-tailed) was used to determine significance, and no significant differences were noted between control and DTG groups for any of the measured metabolites.

To assess whether CEST MRI detects the adverse effects of *in utero* DTG exposure on fetal neurodevelopment, pregnant dams were scanned at GD 17.5. (Figure 1A). T_2_-weighted images were an anatomical reference for embryo brains (Figures 2A–D). The pixel-by-pixel heatmaps of CEST contrast on the representative embryo brains from each study group are presented in Figures 2A,B and Supplementary Figure 2. Representative embryo brains selected to quantitate CEST contrasts at −3.5, 3.5, (Figures 2A,B), and 2 ppm (Supplementary Figure 2) from each study group are identified on a respective high-resolution T_2_-weighted MRI image. In the representative heatmap, an increase in color intensities for CEST effects at both −3.5 and 3.5 ppm was observed in the embryo brains of the DTG group compared to control group. However, color intensity at 2 ppm was comparable between DTG and control groups. Further, the Z-spectra of embryo brains were built from CEST MRI data. An average Z-spectra of control (green) and DTG-treated (red) groups are shown in Figure 2C. In a Z-spectrum, the water proton signal is plotted as a function of saturation frequency, and a CEST effect is presented as a signal drop at a certain saturation frequency. The contrast created by the signal drop is proportional to the concentration of the target molecule. Compared to controls, signal drops were observed at −3.5, and 3.5 ppm in DTG-exposed animals. Signal drops resulted from aliphatic NOE (at −3.5 ppm), and amide and amine protons (at 3.5 ppm) (Wu et al., 2016; Jia et al., 2020; Zhao et al., 2023; Guo and Peng, 2025). Quantitation of CEST signals showed a significant increase of amide and amine CEST effect (p = 0.03) and trend of significance for NOE (p = 0.09) in DTG-exposed embryo brains compared to the controls (Figure 2D). No significant differences were found on amino/amine protons (at 2 ppm) between the control and DTG groups (Figure 2D). These changes at −3.5 and 3.5 ppm indicated that DTG induces metabolomic changes in the embryo brain. Overall, noted hyperintensities in CEST contrasts at NOE and 3.5 ppm reflected cellular membrane lipids and combined effect from mobile proteins or peptides and glutamate, respectively. These CEST data indicate *in utero* DTG-exposure-induced developmental neuronal impairments in the embryo brain (Wu et al., 2016; Zhang et al., 2018; Jia et al., 2020; Zhao et al., 2023; Guo and Peng, 2025).

Further, the same embryo brains were scanned using ^1^H-MRS in the same scanning sessions at GD 17.5 (Figures 2E–G). Representative images of a 3 × 3 × 2.5 mm^3^ voxel on the individual embryo brains utilized for ^1^H-MRS data acquisition are shown in Figure 2E. A representative MRS spectrum presenting successful measurements of metabolites from embryo brain is shown in Figure 2F. Measurable metabolites were alanine (ALA), creatine (CR), glutamate (GLU), glycine (GLY), lactate (LAC), myo-inositol (mI), n-acetyl aspartate (NAA), phosphoryl-choline (PC), taurine (TAU), total choline (tCHO). No significant differences were noted in any measured metabolites between the control and DTG groups (Figure 2G).

### DTG-linked embryo brain metabolomic dysfunction

**FIGURE 3:**
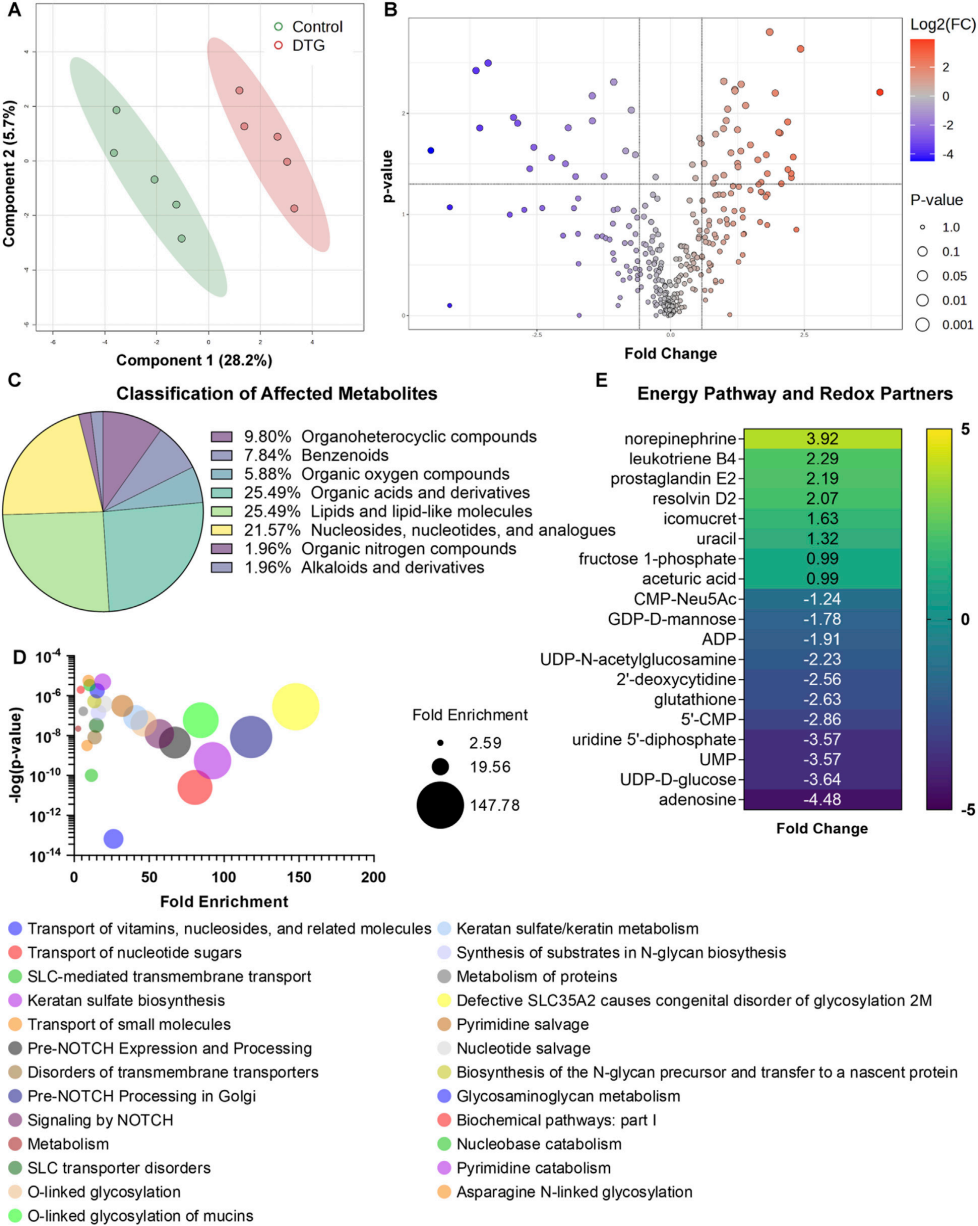
Metabolomic profiling of embryos brains following *in utero* DTG exposure. Global metabolomic profiling of embryo whole brain tissues. Comparison between control and DTG groups were completed, N = 5 animals/group. **(A)** Principal component analysis (PCA) of metabolites. PCA analysis shows 28.2% distribution for control vs. DTG. **(B)** Volcano plot of significantly altered proteins. Red color: Upregulated metabolites; Blue color: Downregulated metabolites; p ≤ 0.05; Absolute Fold Change ≥0.5 or ≤ −0.5. **(C)** Classification of metabolites that were significantly affected by *in utero* DTG-exposure compared to control group. **(D)** Major affected canonical pathways determined by MetaboAnalyst 6.0 and Ingenuity Pathway Analysis (IPA). **(E)** Significantly differentially expressed genes associated with energy pathways and oxidative stress determined by MetaboAnalyst 6.0 and IPA.

Following CEST MRI of live dams, whole brains of embryos were harvested at GD 17.5 (Figure 1A). These were randomly selected from distinct dams of each group for non-targeted metabolomics assessment to determine the effects of *in utero* DTG exposure on the developmental neuro-metabolome. Comparisons of metabolites were completed between control and DTG groups using MetaboAnalyst 6.0. Principal component analysis (PCA) of metabolites showed 28.2% distribution for DTG versus controls confirming group differences (Figure 3A). Metabolite comparisons between DTG-exposed and vehicle-exposed groups showed a total of 54 differentially expressed metabolites. Out of these 19 metabolites were downregulated and 35 were upregulated (Figure 3B; Supplementary Figure 3; Supplementary Table 1). Classification of the differentially expressed metabolites identified 25.49% lipids and lipid-like molecules, 25.49% organic acids and derivatives, 21.57% were nucleosides, nucleotides, and analogues, 9.80% organoheterocyclic compounds, 7.84% benzenoids, 5.88% organic oxygen compounds, 1.96% organic oxygen compounds, and the remaining 1.96% organic nitrogen compounds (Figure 3C). Further analysis using MetaboAnalyst 6.0 and Ingenuity Pathway Analysis (IPA) revealed that differentially expressed metabolites were associated with canonical pathways including energy production, metabolic processes, post-translational modifications, transport pathways, and cell growth and differentiation pathways (Figure 3D). The top 25 affected canonical pathways in the DTG group in comparison to controls are shown in a bubble chart comprising association of p-value and fold enrichment (Figure 3D). Special mentions include transport of nucleotide sugars, SLC-mediated transmembrane transport, pre-NOTCH expression and processing, O-linked glycosylation, keratan sulfate/keratin metabolism or biosynthesis, protein metabolism, and pyrimidine salvage or catabolism. Altogether, these differentially expressed metabolites and affected pathways indicated impairments in cellular processes such as neurogenesis, neuronal growth, dendrite and synapse formation in the embryo brains of the DTG-exposed group compared to the control group. This observation of DTG-induced impairments in cellular processes required for brain development was previously reported (Foster et al., 2023).

We previously reported that DTG treatment induces oxidative stress in the brains of both embryonic and adult mice (Montenegro-Burke et al., 2019; Foster et al., 2023). Therefore, herein, we further extended analysis using IPA to determine whether *in utero* DTG exposure-associated metabolite dysregulation was linked to oxidative stress. Comparative assessment with controls revealed that differentially expressed metabolites in the DTG group were associated with oxidative stress (Figure 3E). In the DTG group, metabolites linked to altered energy production and reactive oxygen species (ROS) production were ADP (adenosine diphosphate), UDP-D-glucose, 5′-CMP (cytidine monophosphate), fructose 1-phosphate, and adenosine. Moreover, glutathione (GSH), a metabolite with antioxidant properties, was reduced. Other notable dysregulated metabolites seen in embryo brains of the DTG group were leukotriene B4, prostaglandin E2, and resolvin D2, indicating inflammation as a consequence of impaired developmental cellular processes (Figure 3E). Altogether, metabolomics assessment indicated *in utero* DTG-induced developmental neuro-metabolome impairments. These results, taken together, support CEST MRI results.

### DTG does not cause neural tube defects (NTDs)

To evaluate the effect of *in utero* DTG exposure on embryo structural development, embryos were harvested at GD 17.5. Embryo phenotypes from the control and DTG-treatment groups are summarized in Table 1. No loss of pregnancy was observed in any group. The litter size, viability, and resorption rates were similar between vehicle and DTG-treatment groups. The mean litter size in the control and DTG groups was 6.6 and 6.8, respectively. The total resorptions in vehicle-treated control dams were 6.1% (n = 5 litters, 2 resorptions out of 33 total implants) and in DTG-treated dams were 14.7% (n = 5 litters, 5 resorptions out of 34 total implants). Out of 31 viable embryos in control and of 29 viable embryos in DTG group, no NTDs (0%) were noted (Table 1). Altogether, *in utero* DTG exposures did not lead to NTDs.

**TABLE 1:** Embryo phenotypes.

	Vehicle	DTG
	Oral	(50 mg/kg/day) oral
Total litters	5	5
Viable pregnancy (%)	100%	100%
Litter Means		
Implants/litter	6.6	6.8
Viable embryos/litter	6.2	5.8
Resorptions/litter	0.4	0.8
Abnormal/litter	0	0
Embryo Phenotype (%)		
Total implants	33	34
Total resorptions	2 (6.1%)	5 (14.7%)
Total viable	31 (93.9%)	29 (85.3%)
Viable Embryos (%)		
Normal	31 (100%)	29 (100%)

### CEST MRI detects fetal brain pathologies and identifies formulation safety profiles

**FIGURE 4:**
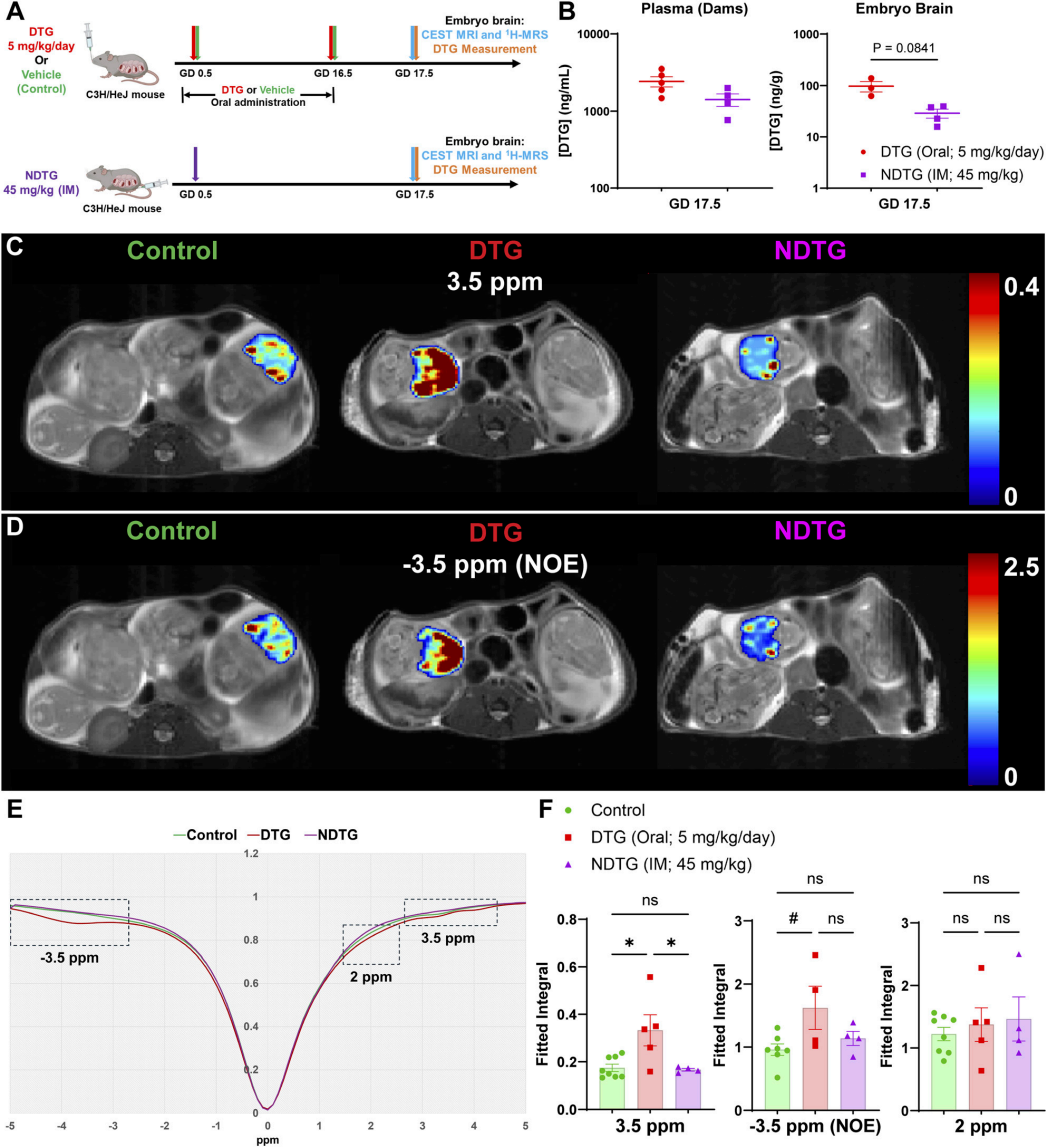
Confirmation of CEST MRI for assessment of drug-induced neuro-metabolomic toxicity and therapeutic strategies. **(A)** Dams were randomly distributed in three study groups. In the first group, dams were daily administered native DTG at 5 mg/kg dose by oral gavage from GD 0.5 to 16.5. In the second group, dams were daily administered with the vehicle by oral gavage from GD 0.5 to 16.5. In third group, dams were injected with a single IM injection of NDTG at 45 mg/kg on GD 0.5. CEST MRI and embryo brain DTG levels were assessed at GD 17.5. **(B)** DTG concentrations in plasma of dams and in whole brain tissues of embryos at GD 17.5. Each biological sample (plasma or tissue) represents a distinct litter. Data are expressed as mean ± SEM, N = minimum 3 animals/group. T-test (two-tailed) was used. **(C,D)** Pixel-by-pixel integral maps of fitted CEST effect. Hyperintensity compared to control was detected in native DTG-treated embryo brains at 3.5 ppm and −3.5 ppm. CEST contrasts for both 3.5 ppm and −3.5 ppm were comparative between control and NDTG groups. **(E)** Z-spectra of controls, native DTG, and NDTG-treated mice. **(F)** Group comparisons of fitted integrals of CEST effect at 2 ppm, 3.5 ppm and −3.5 ppm on embryo brain. N = minimum 4 animals/group. A one-way ANOVA followed by Tukey’s test was utilized to compare each CEST contrast among control, DTG, and NDTG groups (^#^P < 0.1, *P < 0.05; **P < 0.01, ns = not significant).

To verify the reproducibility of CEST MRI in detecting DTG-linked metabolomic changes in the embryo brains, a replicate study was conducted. In this study, the timeline was kept similar to the previous study; however, the treatment scheme differed. This study aimed to evaluate the sensitivity of CEST MRI at a therapeutic dosage of DTG. Moreover, to enhance rigor, an additional group receiving a long-acting nanoformulation of DTG was included. Thus, for this study, dams (C3H/HeJ) were randomly distributed in three study groups, DTG, NDTG, or vehicle (control) (Figure 4A). Similar to what was previously observed (Supplementary Figure 1), no significant differences in body weight gains of dams were observed among control and either of DTG-treatment group (Supplementary Figure 4A), and no NTDs were detected in any of the study groups (Supplementary Table 2). In addition, the effect of native DTG or NDTG exposure on embryo and placental physical growth was assessed. No significant differences were detected for embryo weights, embryo crown-rump length and head diameter, and placenta weights among vehicle, oral native DTG, and IM NDTG groups (Supplementary Figure 4B).

Poloxamer 407 (P407)-DTG nanoformulation (NDTG) was prepared using high-pressure homogenization (Sillman et al., 2018; Foster et al., 2023). Encapsulation efficiency of NDTG formulation was 73.4% (Supplementary Figure 5A). Particle size, polydispersity index (PDI), and zeta potential for the nanoformulation were determined using dynamic light scattering (DLS). The particle size, PDI, and zeta potential of NDTG particles were 298 nm, 0.084, and −23 mV, respectively. Further, SEM evaluation confirmed that NDTG particles possessed uniform rod- and cuboidal-shaped morphologies (Supplementary Figure 5B).

Initially, PK and BD profiles of drug were determined for both native-DTG and NDTG-treated groups by measuring DTG levels in the dam plasma, and in the embryo brain tissue homogenates. These tests were performed to examine our hypothesis that injectable NDTG achieves therapeutic DTG concentrations in maternal blood and, at the same time, limits distribution of DTG to the embryo brain. Single (45 mg/kg) IM injections of NDTG achieved equivalent plasma DTG levels to daily oral DTG administration (5 mg/kg) in pregnant dams. Measured average plasma drug concentrations were 2,428.40 ± 364.8 in DTG-treated dams and 1,412.30 ± 260.4 ng/mL in NDTG-treated dams at GD17.5 (Figure 4B). These plasma DTG levels are comparable to therapeutic DTG concentrations achieved through daily oral dosing in humans (Pain et al., 2015; Lewis et al., 2016; Mulligan et al., 2018; van der Galien et al., 2019). However, lower levels of DTG biodistribution in embryo brains were observed following NDTG injections in comparison to daily oral administration (Figure 4B). For the daily oral native DTG group, an average DTG concentrations of 97.4 ± 22.4 ng/g were noted in embryo brains. Whereas an average of 29 ± 5.8 ng/g of DTG levels were measured in embryo brains of NDTG-treated group (Figure 4B). In addition, an average tissue DTG concentration of 666.7 ± 130.1 ng/g was recorded in placental tissues of the native DTG-treated group compared to 319.3 ± 56.4 ng/g of DTG concentrations in placentas of NDTG-treated group (Supplementary Figure 6). Placenta DTG levels further validated limited transplacental transfer of DTG to embryos in NDTG group compared to native-DTG group. Altogether, PK and BD data confirmed that LA DTG nanoformulations sustain therapeutic DTG levels in maternal plasma and limit overall drug exposure to the embryo brain during pregnancy. The safety benefits of lower biodistribution of drug in embryo brain during gestation were determined in the succeeding *in utero* CEST MRI assessments.

To determine benefits of NDTG in mitigating *in utero* DTG exposure-linked adverse effects on embryo brain metabolites, live pregnant dams were scanned by CEST MRI at GD 17.5. (Figure 4). CEST MRI acquisition and analysis were similar to the first study (Figure 2). The pixel-by-pixel heatmaps of CEST contrasts on the representative embryo brain from each study group are shown in Figures 4C,D. Representative embryo brains selected to quantitate CEST contrasts at 3.5 ppm, 2 ppm, and −3.5 ppm from each study group are identified on a respective high-resolution T_2_-weighted MRI image. Similar to the results from the previous study at a supratherapeutic dosage of DTG (Figure 2), in the representative heatmap, increased color intensity for CEST effects at both 3.5 and −3.5 ppm was observed in the embryo brains of the DTG group at therapeutic dosage compared to that in the embryo brains of vehicle-treated controls. Notably, comparative color intensity was observed at both 3.5 and −3.5 ppm between the control and NDTG-treated groups (Figures 4C,D). Moreover, lower color intensity was noted in embryo brains of the NDTG-treated group than in embryo brains of the native-DTG-treated group (Figures 4C,D). No differences were observed at 2 ppm among vehicle, native-DTG, and NDTG-treated groups (Supplementary Figure 7).

Further, an average Z-spectrum of control (green color), DTG (red color), and NDTG (purple color) mice is shown in Figure 4E. Compared to control animals, signal drops were observed at −3.5 and 3.5 ppm in native-DTG-treated animals. Whereas Z spectra at −3.5 and 3.5 ppm was comparable between the control and NDTG groups. Moreover, signals were comparable for measures of amino and amine protons (2 ppm) among all groups (Figures 4E,F; Supplementary Figure 7). Quantitation of CEST signals showed a significant increase of amide and amine proton CEST effects and trend of significance for NOE effect on embryo brains of native-DTG group compared to the control group (Figure 4F). No significant differences were found in amino/amine effects between the control and native-DTG groups (Figure 4F). Particularly, noted hyperintensities in CEST contrast at both NOE (membrane lipids), and 3.5 ppm (mobile proteins/peptides and glutamate) indicate DTG-linked neuronal impairments in embryo brains. These results for both −3.5 and 3.5 ppm were similar between supratherapeutic dosage and therapeutic dosage of native DTG, confirming reproducibility. Conversely, CEST contrasts at 3.5 ppm were significantly lower in embryo brains of NDTG-treated group compared to those in embryo brains of the native-DTG treatment group. However, these observed CEST values were comparable between control and the NDTG groups. Similar CEST contrasts for all signals (3.5 ppm and −3.5 ppm) between control and NDTG groups signified prevention of DTG-induced developmental neurotoxicity in the embryo brain. In addition, similar to the previous study, the same embryo brains were further scanned using ^1^H-MRS utilizing the same data acquisition and analysis methods. No significant differences were observed for any measured metabolites (ALA, CR, GLU, LAC, mI, NAA, TAU, tCHO) among control, DTG, and NDTG treatment groups (Supplementary Figure 8). Interestingly, the trend of increase was noted in LAC and ALA levels in native DTG group compared to control and NDTG group, respectively, indicating a potential compensatory increase in energy production for neuronal differentiation and growth following injury during early development (Rabah et al., 2023; Wu et al., 2023). Overall, PK, BD, ^1^H-MRS, and CEST MRI data collectively suggest that lower drug exposure to the embryo brains using injectable LA DTG nanoformulations can prevent drug-induced developmental neuronal impairments. Importantly, the reproducibility of the sensitivity of CEST MRI in detecting ARV-induced alterations in embryo brain metabolites was confirmed.

## Discussion

Infants born to mothers LWH remain free of HIV-1 infection due to the benefits of ART. However, in parallel, fetal brains are vulnerable to ARV exposures during gestation. ARVs are known to cross the placental barrier, posing a direct threat to cellular processes occurring during this critical period of neurodevelopment (McCormack and Best, 2014; Rimawi et al., 2017; van der Galien et al., 2019; Cerveny et al., 2021; Foster et al., 2022). Such ARV-induced adverse events that impact early neurodevelopment can influence long-term post-natal neurofunctional outcomes. HEU children exposed *in utero* to ARVs are known to have poor neurodevelopment across multiple domains, including cognitive, motor, language, and socio-emotional functions (Sirois et al., 2013; Caniglia et al., 2016; Cassidy et al., 2019; Crowell et al., 2020; Wedderburn et al., 2022; Bulterys et al., 2023). Still, to date, there is a knowledge gap about whether and how a particular ARV or class thereof may influence early neurodevelopment during pregnancy. Therefore, developing a non-invasive bioimaging tool to identify biomarkers of ARV-linked neuronal impairments or neuroimmune dysfunctions and to support the creation of therapeutic strategies to improve neurodevelopment is timely. Here, we demonstrate for the first time that CEST MRI outperforms traditional ^1^H-MRS in detecting DTG-related impairments in neuronal development.

Poorer neurodevelopmental outcomes in HEU compared to HIV-1 unexposed and uninfected (HUU) children have been recognized in both RRCs and RLCs. Poorer neurodevelopment was noted across multiple domains, but most notably in language and gross motor functions (Wedderburn et al., 2022). Some studies also reported lower academic performance or global cognitive ability in HEU children compared to HUU (Benki-Nugent et al., 2022; Powis et al., 2023). To date, studies have indicated poorer neurodevelopment following gestational exposure to ARVs from different classes including EFV, ATV, DTG and ddI (Sirois et al., 2013; Caniglia et al., 2016; Cassidy et al., 2019; Crowell et al., 2020; Wedderburn et al., 2022; Bulterys et al., 2023). The “Tshipidi Plus” study from Botswana demonstrated an association between *in utero* EFV exposure and lower performance in receptive language, and poorer emotional/behavioral self-regulatory capacities in 2-year-old children (Cassidy et al., 2019). The Surveillance Monitoring for ART Toxicities (SMARTT) study conducted in North America reported that infants with *in utero* EFV exposure had over 50% increased risk of neurologic diagnosis with the most common diagnosis being febrile seizures (Crowell et al., 2020). Gestational exposure to ATV was also associated with poor language outcomes at 12 months of age (Sirois et al., 2013; Caniglia et al., 2016). DTG is now a widely utilized ARV as part of a first line regimen and it recently replaced EFV in RLCs (WHO, 2018; Dorward et al., 2018; Hill et al., 2018; The Lancet, 2020). As usage of DTG is relatively new during pregnancy, very little is known about its effects on neurodevelopmental outcomes. Yet, the SMARTT study, with limited sample size, reported an increased risk of postnatal neurological abnormalities following DTG exposure during development, particularly following exposure at conception or during the first trimester (Crowell et al., 2020), supporting our findings. To date there is no data on neurodevelopmental outcomes following long-acting injectables. Additionally, different potential underlying mechanisms for ARVs-associated neurotoxicity have been proposed. Among these, mitochondrial toxicity and oxidative stress have been commonly reported as potential causal factors for ARVs linked neurotoxicity (Schnoll et al., 2019; Lanman et al., 2021). These ARVs include, but are not limited to, EFV, DTG, and ATV. However, the link between molecular mechanisms and functional neurodevelopmental outcomes still needs to be established. Overall, clinical studies, from both RRCs and RLCs have underscored a high risk of neurodevelopmental impairments following *in utero* ARVs exposure. Nonetheless, the understanding of the underlying mechanisms and early-stage detection means for such toxicity are in their infancy and requires attention.

In the current study, DTG-induced CEST contrast alterations were potentially associated with neuronal membrane lipids, mobile proteins or peptides, and glutamate; all of which are critical for healthy neuronal development including neuronal migration, differentiation, and dendrite or synaptic formation (Olsen and Faergeman, 2017; Hussain et al., 2019; Basu et al., 2021; Oliveira et al., 2022; Viljetic et al., 2024). Global metabolomics data confirmed DTG-associated changes in lipids and metabolites along with associated canonical pathways indicating impairment in cellular processes such as neurogenesis, neuronal growth, dendrite and synapse formation in the embryo brain, supporting CEST MRI findings. Furthermore, changed expression of metabolites such as ADP, UDP-D-glucose, 5′-CMP, fructose 1-phosphate, adenosine, and GSH indicated oxidative stress and altered energy production in the DTG-group. Such DTG-linked oxidative stress has been reported in both embryonic and adult mice brains (Montenegro-Burke et al., 2019; Foster et al., 2023). Dysregulation in these processes can reflect developmental neuronal deficits during gestation predisposing children to functional abnormalities including, but not limited to, language or motor deficits (Olsen and Faergeman, 2017; Hussain et al., 2019; Pregnolato et al., 2019; Basu et al., 2021; Oliveira et al., 2022; Viljetic et al., 2024). These observations of DTG-induced impaired neuronal development in a rodent model were similar to previous reports (Bade AN et al., 2021; Foster et al., 2023). This study further confirmed DTG-associated developmental neuronal deficits at both therapeutic and supratherapeutic dosages. Although CEST MRI data indicated the injury to neuronal development, a direct link to neuronal processes cannot be established through CEST MRI or global metabolomics assessment. In the future, single cell biological assessments will be needed to determine the direct association between CEST data and underlying cell profiles.

Indeed, CEST MRI and ^1^H-MRS are expected to provide complementary insights into the metabolic profile. Though CEST MRI does not allow for direct quantification of specific metabolites, herein, it is proved advantageous over ^1^H-MRS likely driven by its inherently higher spatial resolution and reduced susceptibility to motion artifacts. Nonetheless, in one of the studies, ^1^H-MRS observed a trend of an increase in LAC and ALA levels following exposure to therapeutic DTG dosage, indicating a potential compensatory increase in energy production for neuronal differentiation and growth following drug-induced injury during early neurodevelopment (Rabah et al., 2023; Wu et al., 2023), and supported CEST data, but further studies are needed for definitive conclusions. Despite limited data on the effect of *in-utero* DTG exposure on neurodevelopmental outcomes and no other previous reports studying effects of DTG on metabolites or macromolecules in the fetal brain, it is now well accepted that DTG causes neuropsychiatric adverse events in adults ranging from insomnia to severe depression (Yombi, 2018; Amusan et al., 2020). DTG-associated neuronal toxicity is also confirmed by *in vitro* and research animal studies (Montenegro-Burke et al., 2019; Bade AN et al., 2021; Kirkwood-Johnson et al., 2021; Smith et al., 2022; Foster et al., 2023; Zizioli et al., 2024). Thus, with the transition to DTG-based regimens as a recommended first-line therapy worldwide and reported DTG-linked neuronal toxicity, it is essential to identify the effects of *in utero* DTG exposure on neurodevelopment, uncover underlying mechanisms, as well as develop novel therapeutic interventions to maximize therapeutic gains of DTG while limiting adverse events during pregnancy.

Herein the rigor and reproducibility of CEST MRI in detecting embryo brain metabolites was confirmed by affirming the benefits of long acting nanoformulations of DTG (NDTG) in preventing drug-associated metabolite alterations. Previously, we evaluated the safety and benefits of NDTG against daily oral native DTG administration in pregnant mice. Similar to the current study, previous work demonstrated that NDTG reduces native DTG-associated impairment of neuronal development in the embryo brain (Foster et al., 2023). This neuroprotective effect is the outcome of measured lower drug biodistribution in the embryo brain following NDTG injection in comparison with daily oral DTG administration. Lower drug biodistribution to embryo brains is predicted to be due to the pharmacological properties of NDTG, including a longer half-life and dosing intervals, maintenance of a long-term lower C_max_, and enhanced bioavailability. These enhanced PK properties are due to sustained release of DTG from the formulation and tissue and cell depots of formulations (Montenegro-Burke et al., 2018; Sillman et al., 2018). Moreover, the poloxamer surfactant utilized for nanoformulations is expected to provide supplemental therapeutic benefits as poloxamers are neuroprotective, anti-inflammatory, and antioxidative (Curry et al., 2004b; Curry et al., 2004a; Serbest et al., 2006; Cadichon et al., 2007; Dalal and Lee, 2008; Moloughney and Weisleder, 2012; Luo et al., 2013; Luo et al., 2015). Poloxamers are also known to promote resealing of disrupted plasma membranes (Curry et al., 2004b; Curry et al., 2004a;Serbest et al., 2006; Cadichon et al., 2007; Dalal and Lee, 2008; Moloughney and Weisleder, 2012; Luo et al., 2013; Luo et al., 2015). The selection of a P407-based formulation for this study was based on our own work that previously showed the benefits of P407 encapsulated DTG nanoformulations in attenuating DTG-associated impaired neuronal development in mice embryo brain (Foster et al., 2023) and reducing oxidative stress in the central nervous system (CNS) of adult mice (Montenegro-Burke et al., 2018). The current study shows the strength of CEST MRI in screening different therapeutic means, formulations, or additive strategies to elicit improved safety profiles of the potent and broadly utilized DTG and potentially other ARVs during pregnancy.

We did not observe any type of neural tube defects (NTDs) or other structural abnormalities in native DTG at both therapeutic and supratherapeutic dosages or in NDTG-treatment group. Although initial interim analysis of Tsepamo study from Botswana reported potential risk if NTDs in infants exposed to DTG periconceptionally, such risk of chronic congenital malformation has diminished according to a recent update (the 24th International AIDS Conference, 2022). These findings supporting no risk of NTDs following DTG exposure at conception are consistent with our observations from previous works at both therapeutic and supratherapeutic dosages in rodent models (Bade AN et al., 2021; Foster et al., 2023).

The World Health Organization (WHO) recommends ART for pregnant women LWH in both RRCs and RLCs (WHO, 2016; DHHS, 2025b). Worldwide, more than 15 million women of child-bearing age are living with HIV-1 infection, and in 2023, every week, 4000 adolescent girls and young women aged 15–24 years became HIV-1 infected in sub-Saharan Africa (UNAIDS, 2024). Due to improved coverage of ARVs, particularly in RLCs, the proportion of pregnant women LWH who conceive on ART is increasing rapidly. Thus, the population of HEU children born following *in utero* exposure to ARVs has grown to nearly 16 million (Cassidy et al., 2019; UNAIDS, 2023; Bulterys et al., 2023; Wedderburn et al., 2024). Furthermore, in some RLCs, over 20% of children (<15 years age) are born to mothers LWH (UNAIDS, 2023; Bulterys et al., 2023; Wedderburn et al., 2024). However, despite the substantial benefits of ART in preventing HIV-1 transmission to fetus during pregnancy, there are risks of adverse events on neurodevelopment linked to gestational exposure to ARVs (Hill et al., 2018). There is a research gap regarding known effects and underlying mechanisms that reflect ARV-induced neurodevelopmental impairments in children born without any form of chronic structural malformations. Therefore, with over a million HEU infants born each year (Ramokolo et al., 2019; Crowell et al., 2020), it is important to assure their safety during development. To achieve this goal, the development of a highly sensitive non-invasive bioimaging tool like CEST MRI for early recognition of ARV-associated adverse events during pregnancy and for uncovering altered molecular biomarkers as underlying mechanisms remains timely.

In recent years, integrase strand transfer inhibitors (INSTIs) use has become widespread. INSTIs are part of preferred first- and second-line ART regimens (WHO, 2016; European AIDS Clinical Society, 2017; DHHS, 2025a; DHHS, 2025b), as ARVs from this class are known for high efficacy in terms of rapid viral suppression and barriers to drug resistance (Smith et al., 2021). In particular, the availability of DTG-based regimens has substantially increased in the past 5 years. Due to a pricing agreement made in 2017, more than 100 RLCs transitioned to a DTG-based generic regimen by mid-2020 (The Lancet, 2020; Fairhead et al., 2024). In 2019, ∼6.9 million people had access to this generic DTG-regimen, and up to 15 million people LWH worldwide were expected to be treated with DTG by the year 2025 (WHO, 2018; Dorward et al., 2018; Hill et al., 2018; The Lancet, 2020). This includes women of child-bearing age, who remain a significant population LWH (UNAIDS, 2024). Moreover, increasing pretreatment drug resistance to non-nucleoside reverse transcriptase inhibitors (NNRTIs) in RLCs, especially in women, expands usage of DTG-based regimens (WHO, 2019a; WHO, 2019b). Thus, due to current inclusion of DTG-based regimen in recommended guidelines for treatment of pregnant women or those of child-bearing potential worldwide due to its high benefits to risk ratio (WHO, 2019b; DHHS, 2025b) and rising pretreatment resistance to NNRTIs in RLCs, a majority of women LWH are and will be treated with DTG-based regimens globally. Apart from DTG, bictegravir (BIC) is currently recommended as “preferred” during pregnancy, and long-acting cabotegravir (LA-CAB) injectable is currently “not recommended” due to lack of data on safety, dosing, and PK during pregnancy (DHHS, 2025b). However, due to their (BIC and CAB) known high efficacy in adults, especially in cisgender women (Orkin et al., 2021; Delany-Moretlwe et al., 2022), their usage in pregnant women at a large scale is inevitable. Clinical reports of pharmacological events associated with INSTIs have shown that they carry a class effect. Thus, in addition to DTG, studies focused on determining effects of other INSTIs as class effect on fetal neurodevelopment would be needed in the future.

Although the current study is strengthened by rigor and reproducibility and showcases that CEST MRI can detect DTG-induced neuro-metabolite alterations indicating developmental neuronal impairments in mouse embryo brains, a few limitations were recognized. We acknowledge that CEST MRI cannot distinguish the effect of ARVs on neuro-metabolome from other co-morbidities like exposure to viral proteins or drugs of abuse. Once validated, CEST MRI may be able to detect ART influence on region specific metabolites in a pre-clinical model. This knowledge can help researchers to improve their understanding of the role of ARVs on observed neurodevelopmental impairments and to study new ARVs or those in development. Another noted limitation is the absence of a poloxamer (P407; vehicle) IM injection only group as a control. Though, previously, no confounding effects from the poloxamer only group was noted (Montenegro-Burke et al., 2019). Further, mothers are HIV-1 positive in clinics, and such maternal infection, even at an undetectable level, could be a confounding factor. In pre-clinical research, normal mice do not get infected with HIV-1; and humanized mice susceptible to HIV-1 infection cannot breed. Thus, to establish cause and effect relationship with ARVs alone, in this study toxicity was evaluated in normal mice. This major limitation linked to testing HIV-1-related confounding mechanisms on neurodevelopment remains in preclinical studies. Currently, the CEST data is acquired on a single slice positioned at the center of the embryo brain. Future efforts including AI models will be needed to develop a 3D multi-slice CEST sequence with varying radio frequency (RF) saturation power that enables the simultaneous acquisition of different metabolites from the entire brain with rapid acquisition (Bhattarai et al., 2025). Such an approach not only will allow studying global neuropathology but also will provide an opportunity to perform brain region specific assessments. Furthermore, while global metabolomics data confirmed CEST findings, a direct correlation between CEST contrast at a saturation frequency and a particular metabolite, protein or peptide alteration was not observed. Identifying metabolites and macromolecules associated with neuronal processes and potential region-specific changes through improved CEST MRI acquisition methods in the future will enhance the ability to predict postnatal neurofunctional outcomes.

*In toto*, ART is the only successful option, currently, for treatment or prevention therapies for people LWH or at risk of acquiring HIV-1. Thus, significant efforts have been ongoing to develop new potent ARVs or long acting injectables. With more than a million ARVs-exposed HEU infants born each year (Ramokolo et al., 2019; Crowell et al., 2020) and continuous efforts to develop new ARVs, development of a non-invasive bioimaging tool which can help for early-recognition of ARV-linked adverse neurodevelopmental outcomes is needed now more than ever. Although the current study focused on a DTG-based model due to known toxicity, the developed CEST MRI tool can be employed to study the effect of other clinically approved or in development ARVs. This is due to the specificity of CEST MRI to developmental cellular mechanisms and not to a type of ARV. CEST MRI will identify ART-induced neuronal health-linked metabolomic alterations in embryo brain as underlying mechanisms for neurodevelopmental deficits. Detection will be irrespective of the classification of ARVs or administration route. Due to its non-invasive nature, the successful implementation of CEST MRI to study neurodevelopment in rodent models will provide a tool with a translational potential for early-stage biomarker discovery.

## Data Availability

The original contributions presented in the study are included in the article/Supplementary Material, further inquiries can be directed to the corresponding authors. In addition, the metabolite profiling data (metabolomics) presented in the study are deposited in the figshare repository, accession number https://doi.org/10.6084/m9.figshare.30172402.v1.
