# Supplementary material for: CEST MRI detects antiretroviral drug toxicities in the developing mouse brain
Source: Front Pharmacol. 2025 Oct 29;16:1681094. doi: 10.3389/fphar.2025.1681094 (PMC12605090; doi:10.3389/fphar.2025.1681094)
Supplement: Supplementary file 1 [file DataSheet1.pdf]

Supplementary Material

CEST MRI Detects Antiretroviral Drug Toxicities in the Developing Mouse Brain

Micah Summerlin[1], Mariano G. Uberti[2], Dhananjay Shinde[3], Emma G. Foster[1], Brady Sillman[1], Manjeet Kumar[1], Baojin Yao[1], Dongming Peng[4], Benson J. Edagwa[1], Howard E. Gendelman[1,5], Yutong Liu[1,2]*, Aditya N. Bade[1]*

[1]Department of Pharmacology and Experimental Neuroscience, University of Nebraska Medical Center, Omaha, NE 68198, USA.

[2]Department of Radiology, University of Nebraska Medical Center, Omaha, NE 68198, USA.

[3]Department of Pathology, Microbiology and Immunology, University of Nebraska Medical Center, Omaha, NE 68198, USA.

[4]Department of Electrical and Computer Engineering, University of Nebraska – Lincoln, Lincoln, NE, 68508, USA.

[5]Department of Pharmaceutical Sciences, University of Nebraska Medical Center, Omaha, NE 68198, USA.

***Corresponding authors:**

Aditya N. Bade, Ph.D., Department of Pharmacology and Experimental Neuroscience, University of Nebraska Medical Center, Omaha, NE 68198-5800, USA; phone: 402-559-4050; fax: 402-559-7495; email: aditya.bade@unmc.edu, ORCID: https://orcid.org/0000-0003-2511-4461 (for submission, contact, and correspondence);

Yutong Liu, PhD; Department of Radiology, University of Nebraska Medical Center, Omaha, NE, 68198-1045, USA; phone: 402-559-4050; fax: 402-559-4051; email: yutongliu@unmc.edu, ORCID: https://orcid.org/0000-0002-3797-8681 (for correspondence)

Supplementary Figures

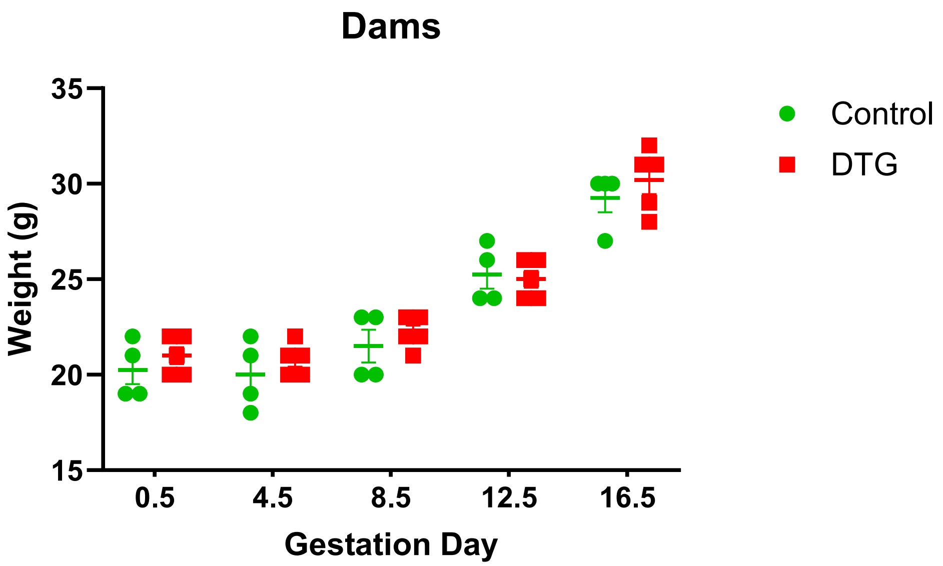

Supplementary Figure 1. Pregnant mice (dams) weight gain during gestation. Weight gain of dams was recorded during gestation and no significant differences were observed between control and DTG groups. Data are expressed as mean ± SEM, N = minimum 4 animals/group. t test (two-tailed) was used to determine significant differences in weight gain between control and DTG group. No significant differences were observed.

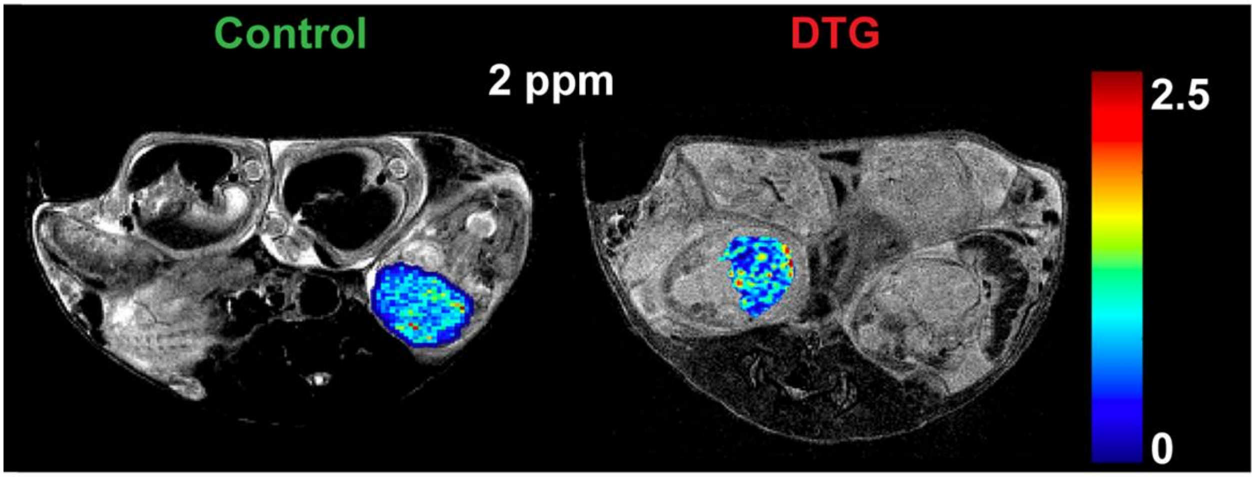

Supplementary Figure 2. CEST MRI on embryo brain at 2 ppm. Live pregnant dams (C3H/HeJ) were scanned at GD 17.5 using a 7 T scanner. Pixel-by-pixel integral maps of fitted CEST effect. Comparative intensity was detected in embryo brains between both study groups, control and DTG, at 2 ppm.

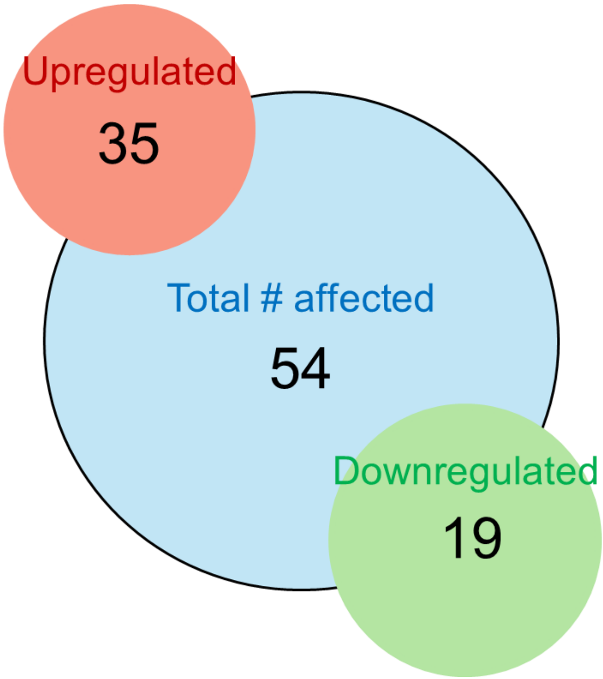

Supplementary Figure 3. Metabolomic profiling. Schematic presentation of total number of affected metabolites including upregulated and downregulated metabolites in DTG-exposed embryo brains compared to controls. MetaboAnalyst 6.0 was utilized for assessment. N = 5 animals/group.

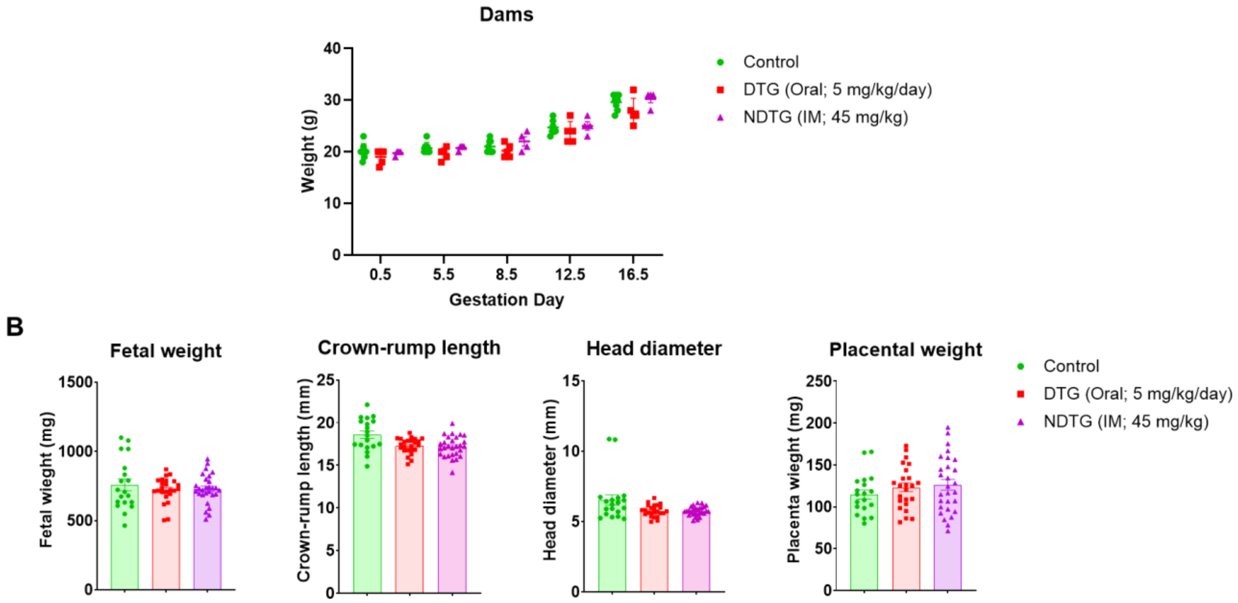

Supplementary Figure 4. Maternal body weight gain during gestation and embryo phenotypes. (**A**) Weight gain of dams was recorded during gestation and no significant differences were observed among control (N = 8), DTG (N = 5), and NDTG (N = 4) groups. (**B**) Embryo and placenta phenotypes. Placenta weights and embryo weights, crown-rump length and head diameter were measured. No significant differences were observed among control, DTG, and NDTG groups.

A

Size	298 nm
PDI	0.084
Zeta Potential	-23 mV
Encapsulation efficiency	73.4%

B

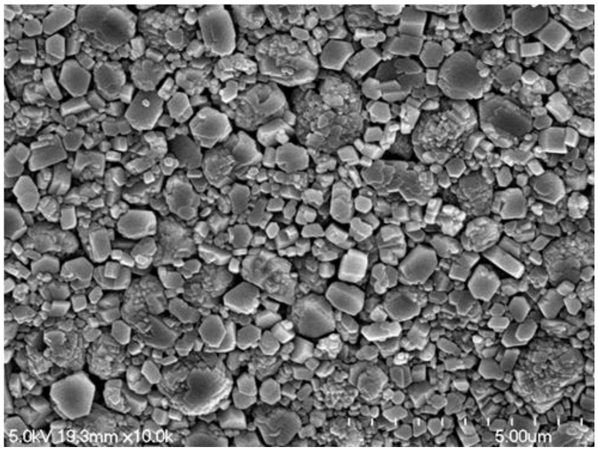

Supplementary Figure 5. NDTG nanoparticle characterization. (**A**) NDTG particles were characterized for hydrodynamic particle diameter (size), polydispersity indices (PDI), and zeta potential using a Malvern Zetasizer Nano-ZS. Drug content of formulation was measured using UPLC-TUV. (**B**) Morphological assessment of NDTG particles was performed using scanning electron microscopy (SEM).

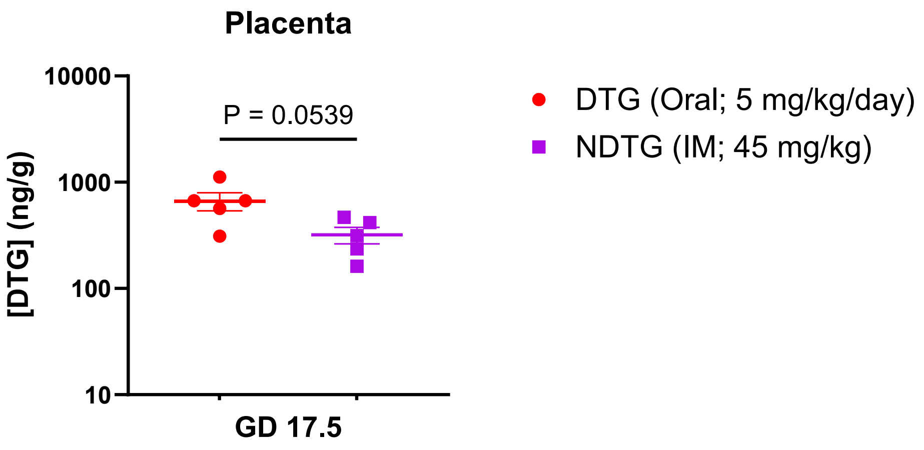

Supplementary Figure 6. Biodistribution of DTG to placenta. Drug concentrations in placenta at GD 17.5 were measured. Data are expressed as mean ± SEM, N = minimum 4 animals/group. t test (two-tailed) was used to compare DTG levels in placenta tissues between DTG and NDTG groups. (*P < 0.05, #P < 0.1).

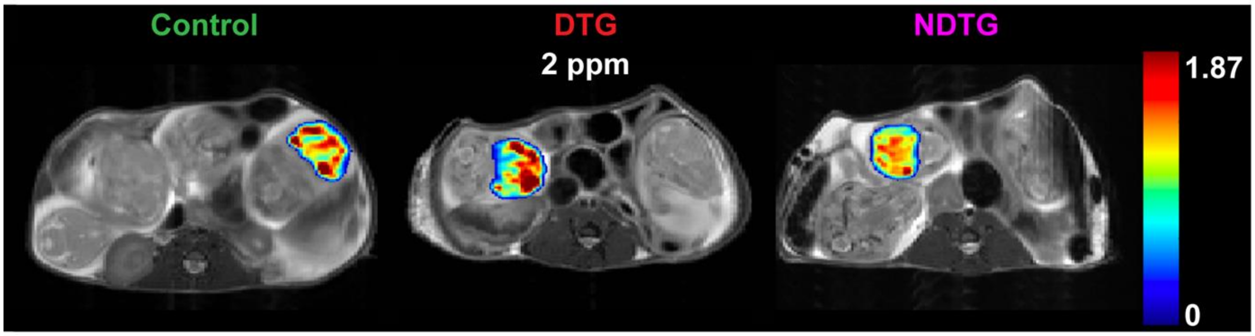

Supplementary Figure 7. CEST MRI on embryo brain at 2 ppm. Live pregnant dams were scanned at GD 17.5. Comparative color intensity was detected in embryo brains among all three study groups (control, native DTG, and NDTG) at 2 ppm.

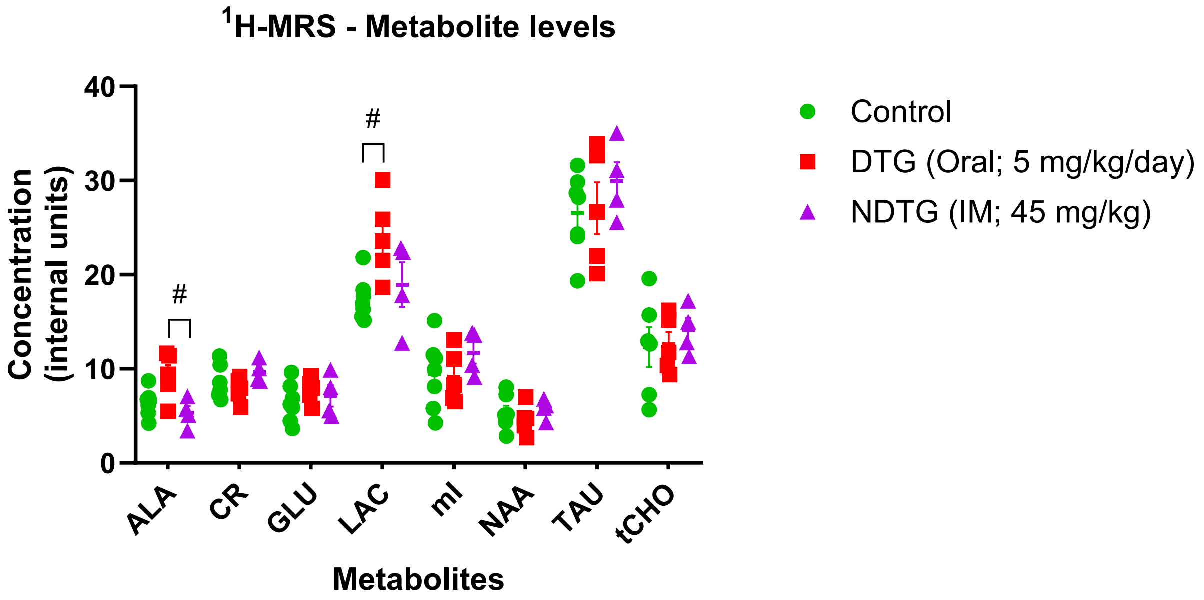

Supplementary Figure 8. ^{1}H-MRS on embryo brains. Metabolite concentrations (mean ± SEM) in embryo brains are expressed as an internal units (i.u.) using ^{1}H-MRS scans. Data are expressed as mean ± SEM , N = minimum 4 animals/group. A two-way ANOVA followed by Tukey's test was utilized to compare each metabolite among control, DTG, and NDTG groups ($^{\#}P < 0.1$).

Supplementary Tables

Supplementary Table 1. Global metabolomic profiling.

Metabolites	FC	log2(FC)	raw.pval	(-LOG10(p))
Ethylparaben	3.6117	1.8527	0.00157	2.8032
Furaneol	5.3866	2.4294	0.00231	2.6362
Phytosphingosine	0.0938	-3.4142	0.00319	2.4968
Uridine diphosphate glucose	0.0802	-3.6403	0.00378	2.4225
Fructose 1-phosphate	1.9911	0.99355	0.00483	2.3158
2-Dodecylbenzenesulfonic acid	0.47815	-1.0645	0.00491	2.3087
Uracil	2.4938	1.3183	0.00516	2.287
N-[(6-Hydroxy-1,3-benzodioxol-5-yl)carbonyl]glycine	2.2974	1.2	0.00583	2.2342
L-Dopachrome	2.3012	1.2024	0.006	2.2216
noradrenaline	15.082	3.9148	0.0062	2.2079
Val-Ser	3.8803	1.9562	0.0063	2.2004
N(2)-succinyl-L-ornithine	0.36192	-1.4663	0.00672	2.1725
Malondialdehyde	2.6421	1.4017	0.00838	2.0768
N-(2,3,4,5-Tetrahydroxybenzoyl)glycine	2.0936	1.066	0.0091	2.0409
L-Asparagine	0.60088	-0.7348	0.00931	2.0311
5-BETA-D-RIBOFURANOSYLPICOLINAMIDE ADENINE-DINUCLEOT	0.13036	-2.9394	0.01098	1.9596
N-[3-Carboxy-2-(carboxymethyl)-2-hydroxypropanoyl]glutamic acid	2.0565	1.0402	0.01182	1.9273
Adenylthiomethylpentose	0.36255	-1.4638	0.01189	1.9247
12(13)Ep-9-KODE	4.5615	2.1895	0.01216	1.9149
Cytidine 5'-monophosphate	0.13756	-2.8619	0.01253	1.9021
Adenosine diphosphate	0.26532	-1.9142	0.01391	1.8568
Uridine 5'-diphosphate	0.08423	-3.5696	0.01397	1.8548
Aceturic acid	1.9831	0.98777	0.01418	1.8483
2,4-Dihydroxy-7,8-dimethoxy-1,4-benzoxazin-3-one	2.3806	1.2513	0.01454	1.8374
Butylated hydroxytoluene	4.0643	2.023	0.01546	1.8109
2-Hexenoylcarnitine	4.1284	2.0456	0.01567	1.8049
Linamarin	1.791	0.8408	0.01607	1.7941
15,16-DiHODE	2.8124	1.4918	0.02055	1.6872
2'-Deoxycytidine	0.16975	-2.5585	0.02169	1.6638
Adenosine	0.04468	-4.4842	0.02331	1.6324
N(1)-acetylspermidine	0.5582	-0.8412	0.02357	1.6277
Dimethyluric acid	2.4842	1.3128	0.02364	1.6263
LU0875000	2.1852	1.1277	0.02459	1.6093
Glyoxylic acid	0.63326	-0.6591	0.02572	1.5897
8,9-DiHETrE	3.4539	1.7882	0.02574	1.5894
Leukotriene B4	4.8997	2.2927	0.02712	1.5668
UDP-GlcNAc	0.21389	-2.2251	0.02748	1.5611
icomucret	3.0945	1.6297	0.02887	1.5396
RG1300000	2.3818	1.252	0.03129	1.5047
2,4,6-Trimethyl-1,3,5-dithiazinane	1.5452	0.62776	0.03138	1.5033
1-oleoyl-2-arachidonoyl-sn-glycerol-3-phosphoethanolamine	0.25574	-1.9673	0.03154	1.5012
CYS-ASP	2.396	1.2606	0.03391	1.4696
Reduced Glutathione	0.16108	-2.6341	0.03534	1.4517
Prostaglandin E2	4.5709	2.1925	0.03593	1.4446
2,3-Dihydroxypropyl beta-D-galactopyranoside	4.7737	2.2551	0.0393	1.4056
2-(N(Omega)-L-arginine)succinic acid	3.5042	1.8091	0.03948	1.4037
Maleic acid	1.9911	0.99357	0.04096	1.3876
CMP-Neu5Ac	0.42219	-1.244	0.04189	1.3779
GDP-alpha-D-mannose	0.2916	-1.7779	0.04219	1.3748
DL-Carbocysteine	4.7818	2.2576	0.0433	1.3636
3-oxopalmitic acid	1.7907	0.84056	0.0433	1.3635
A-12(13)-EpODE	3.233	1.6929	0.04808	1.318
8-Methyl-8-azabicyclo[3.2.1]octane-1,2,3,4,6-pentol	1.7379	0.79738	0.04919	1.3081
Resolvin D2	4.202	2.0711	0.04961	1.3044

Supplementary Table 2. Embryo phenotype characterization.

	Vehicle Oral	DTG (5 mg/kg/day) Oral	NDTG (45 mg/kg) IM injection
**Total litters**	8	5	4
**Viable pregnancy (%)**	100%	100%	100%
**Litter Means**			
**Implants/litter**	7.38	6.8	8
**Viable embryos/litter**	6.25	4.8	7
**Resorptions/litter**	1.13	2	1
**Abnormal/litter**	0	0	0
**Embryo Phenotype (%)**			
**Total implants**	59	34	32
**Total resorptions**	9 (15.3%)	10 (29.4%)	4 (12.5%)
**Total viable**	50 (84.7%)	24 (70.6%)	28 (87.5%)
**Viable Embryos (%)**			
**Normal**	50 (100%)	24 (100%)	28 (100%)
